# Risk factors predicting residual lesion in subsequent hysterectomy following cold knife conization (CKC) for high-grade squamous intraepithelial lesion (HSIL)

**DOI:** 10.1186/s12905-022-01939-z

**Published:** 2022-08-30

**Authors:** Yong Zeng, Tao Jiang, Yahong Zheng, Jing Yang, Hua Wei, Cunjian Yi, Yan Liu, Keming Chen

**Affiliations:** 1 Department of Obstetrics and Gynecology, The First Affiliated Hospital of Yangtze University, 8 Hangkong Road, Shashi District, Jingzhou, Hubei China; 2 Department of Neuro-Care Unit, The First Affiliated Hospital of Yangtze University, Jingzhou, Hubei China

**Keywords:** Residual lesion, HSIL, CKC, Hysterectomy

## Abstract

**Objective:**

To determine risk factors predicting residual lesion in a subsequent hysterectomy follow a cold knife conization (CKC) for high-grade squamous intraepithelial lesion (HSIL).

**Method:**

Between January 2010 and December 2021, a total of 740 patients who underwent a hysterectomy within 3 months after CKC for HSIL were included in this study. We analyzed their demographic features and pathological parameters. A logistic regression model was used to analyze the relationship between parameters and residual lesion in subsequent hysterectomy specimens.

**Results:**

104 (14.1%) had residual lesion in the hysterectomy specimen, 3 patients with microinvasive carcinoma. The rate of residual lesion in patients with positive endocervical margin was 31.3%, with positive ectocervical margin was 15.3%, with positive combine margin was 38.6%. In multivariate analysis, positive margin (OR 4.015; 95% CI 2.526–6.381; *P* < 0.001), glandular involvement (OR 3.484; 95% CI 1.457–8.330; *P* = 0.005), HPV16/18 infection (OR 2.804; 95% CI 1.705–4.611; *P* < 0.001) and multiple HR-HPV infection (OR 1.813; 95% CI 1.130–2.909; *P* < 0.014) were independent risk factors for residual lesion. The AUC calculated by logistic regression model was 0.78.

**Conclusion:**

Positive margin, positive glandular involvement, HPV16/18 and multiple HR-HPV infection were independent high risk factors of residual lesion in a subsequent hysterectomy following CKC for HSIL.

## Introduction

High-grade squamous intraepithelial lesion (HSIL) was currently recognized as a precancerous lesion of cervical cancer. Without active treatment, approximately 31–50% of patients can progress to cervical cancer within 30 years, the risk of cancer can be reduced to 0.7% after routine treatment [1]. Cervical conization was currently the standard operation for HSIL, including cold knife conization (CKC) and annular loopelectrical excision procedure (LEEP). The purpose of treatment was to completely remove the lesion and prevent canceration, to avoid missed early or latent cervical cancer. However, studies had reported residual lesion of HSIL after conization ranges from 5 to 60% [2–4]. Residual lesion was a risk factor for disease recurrence and progression. With the opening of the third-child policy in China and the delayed age of female childbearing, the treatment of HSIL has brought many challenges, and undertreatment or overtreatment often lead to various complications. Therefore, it was particularly important to find the high-risk factors related to residual lesion, timely treatment intervention for patients with suspected residual lesion after cervical conization, and individualized treatment based on standardization.This study retrospectively analyzed the clinical characteristics of patients with hysterectomy after CKC for HSIL in our hospital in the past 10 years, and summarized the risk factors of postoperative residual lesion.

## Materials and methods

The clinicopathological data of patients who underwent hysterectomy after CKC for HSIL at the department of Obstetrics and Gynecology of the First Affiliated Hospital of Yangtze University between January 2010 and December 2021 were collected. This study was approved by the Ethics Committee of the First Affiliated Hospital of Yangtze University. Informed consent was obtained for all patients or family members.

740 patients were considered eligible for current study if they fulfilled the following inclusion criteria: ① preoperative colposcopy was performed to assess the nature and scope of the lesion, cervical pathological biopsy was HSIL, ② CKC was performed at the initial treatment, ③ the pathological examination after CKC was still HSIL, ④ patients who underwent further hysterectomy within 3 months after CKC, ⑤ complete clinicopathological data were available.

Indications of CKC included HSIL, colposcopy suspected invasive carcinoma, colposcopy was not satisfactory, cervical cytology was inconsistent with colposcopy/histology, endocervical curettage pathological results were positive.

Reference guidelines for indications of hysterectomy: 2006 edition of Chinese expert opinion include: middle-aged and elderly cervical intraepithelial neoplasia (CIN) III patients who had no fertility requirements, CIN III patients who had no fertility requirements complicated with other benign gynecological diseases, and CIN III patients who had completed fertility requirements with positive margins [5]. 2020 edition of Chinese expert consensus: repeated cervical conization with residual lesion in HSIL was difficult, HSIL combined with other gynecological diseases had surgical indications, patients with relapse and unable to perform conization after treatment, and patients with poor compliance after cervical conization [6].

Referring to the standardised nomenclature protocol for cervical lesion jointly published in 2012 by the American Society for Pathology (CAP) and the American Society for Colposcopy and Cervical Pathology (ASCCP), cervical intraepithelial lesion was classified as follows: HSIL and LSIL. HSIL includes CIN 2 and 3 [7]. Patients with cervical intraepithelial lesion were reclassified according to this protocol.

Patients with HSIL or above lesion in hysterectomy specimens were considered as residual lesion. The presence of LSIL alone was not regarded as residual lesion.

Margin status of conization specimens was considered to be positive if cervical intraepithelial neoplasia 1–3 were found in the resection margin of about 1 mm or less. Including:endocervical margin, ectocervical margin and combined margin. Combined margin positive means both endocervical and ectocervical margin were positive.

Lesion range: the cervix was divided into four quadrants and the lesion range was determined according to the number of quadrants involved.

HPV (human papillomavirus) Test: HPV subtypes were detected in the reproductive tract shed cells by microarray technology, using microdot sampling technique, HPV type specific probe was applied to the matrix of gene chip; then PCR amplification, hybridization and coloration were performed on the DNA in samples; Finally, the HPV typing gene chip detection reading system automatically collects images and analyzes and reports the detection results. Multiple HR-HPV infection was defined as two or more HR-HPV infections.

### Statistical analysis

SPSS20.0 software was used for statistical analysis. The count data were expressed as absolute numbers and percentages (%) using *X*^2^ test or Fisher's exact probability method. The measurement data conforming to normal distribution by normality test were expressed as mean ± standard deviation and t test was adopted. Those that do not fit the normal distribution was represented by the median (interquartile) and rank sum test was used. Logistic regression model was used to analyze the influencing factors of residual lesion in uterine specimens. The predictive value was evaluated according to the area under receiver operator characteristic (ROC) curve (AUC) calculated by Logistics regression model. AUC of 0.9–1 indicated that the predictive value of this indicator was very high. AUC of 0.7–0.9 indicates good predictive value, AUC of 0.5–0.7 indicates average predictive value, and AUC < 0.5 indicates no predictive value. *P* < 0.05 was considered as statistically significant.

## Results

740 patients who underwent hysterectomy after CKC for HSIL, the average age was 47.9 years, and median age was 47 years (range 30–78), 254 patients (34.3%) was 50 years or older, and 201 patients (27.2%) was postmenopausal. There were 581 patients (78.5%) with education level below high school, and most of the patients were poorly educated. There was also a slight difference in the administrative region, the rural population accounts for 58% and urban population 42%. Only 16.9% of patients needed surgery for other gynecological diseases. HR-HPV infection was detected in the vast majority of patients, 690 (93.2%) HR-HPV infection, 408 (55.1%) HPV16/18 infection, and 191 (25.8%) multiple HR-HPV infection. 610 patients (82.4%) had abnormal Thin-cytologic test (TCT), including 113 patients (15.3%) with HSIL, 100 patients (13.5%) with ASC-H, 49 patients (6.6%) with LSIL, and 348 patients (47%) with ASCUS, which suggest that HPV/TCT plays an important role in cervical screening. According to the results of preoperative colposcopy, 409 cases (55.3%) had cervical lesion in 3 or more quadrants. According to the pathological result of CKC, glandular involvement were involved in 568 patients (76.8%), 254 patients (34.3%) had positive resection margin, including 112 endocervical margin positive, 85 ectocervical margin positive and 57 combine margin positive. 104 patients (14.1%) had residual lesions in their hysterectomy specimen, including HSIL in 101 patients and 3 patients with microinvasive carcinoma (Tables 1, 2).

**Table 1:** Characteristics of patients with microinvasive carcinoma

Patients	Age	Combine margin	Glandular involvement	HPV	TCT	Lesion range
1	44	Positive	Positive	HPV-16/18 infection	HSIL	4
2	55	Positive	Positive	Multiple HR-HPV infection	ASCH	2
3	36	Positive	Positive	HPV-16/18 infection	HSIL	3

*HR-HPV* high-risk human papillomavirus, *TCT* thin-tytologic test, *HSIL* high-grade squamous intraepithelial lesion, *ASCH* atypical squamous cells, cannot exclude HSIL

**Table 2:** Univariate analyses for prediction of residual lesion in subsequent hysterectomy specimen

Parameter	Numbers		OR (95%CI)	*P*-values
	Total	Residual lesion (%)		
*Age*				
≥ 50	254	42 (16.5%)	1.355 (0.886–2.072)	0.160
< 50	486	62 (12.8%)		
*Menopause*				
Yes	201	35 (17.4%)	1.436 (0.922–2.238)	0.108
No	539	69 (12.8%)		
*Smoking*				
Yes	13	3 (23.1%)	1.959 (0.503–6.872)	0.409
No	727	101 (13.9%)		
*Diseases of the immune system*				
Yes	64	9 (14.1%)	1.001 (0.479–2.092)	0.998
No	676	95 (14.1%)		
*Gravidity*				
≥ 3	506	67 (13.2%)	0.813 (0.526–1.256)	0.349
< 3	234	37 (15.8%)		
*Parity*				
≥ 2	312	45 (14.4%)	1.054 (0.694–1.602)	0.805
< 2	428	59 (13.8%)		
*Lesion range*				
≥ 3	409	69 (16.9%)	1.716 (1.110–2.653)	0.014*
< 3	331	35 (10.6%)		
*Cone margin status*				
Positive	254	70 (27.6%)	5.058 (3.244–7.885)	< 0.001*
Negative	486	34 (7%)		
*Glandular involvement*				
Positive	568	98 (17.3%)	5.769 (2.483–13.404)	< 0.001*
Negative	172	6 (3.5%)		
*HPV-16/18 infection*				
Positive	408	79 (19.4%)	2.949 (1.832–4.746)	< 0.001*
Negative	332	25 (7.5%)		
*Multiple HR-HPV infection*				
Positive	191	38 (19.9%)	1.818 (1.172–2.818)	0.007*
Negative	549	66 (12%)		
*Lesion level*				
CIN II	58	6 (10.3%)	0.688 (0.288–1.644)	0.397
CIN III	682	98 (14.4%)		

*HR-HPV* high-risk human papillomavirus, *OR* odds ratio, *CI* confidence interval, *CIN* cervical intraepithelial neoplasia,

*Statistically significant

Correlation analysis of demographic and clinicopathological parameters related to residual lesion in post-cone hysterectomy specimen. Univariate analysis showed that positive margin (*P* < 0.001), glandular involvement (*P* < 0.001), lesion range (*P* = 0.014), HPV-16/18 infection (*P* < 0.001), and multiple HR-HPV infection (*P* = 0.007) were significantly associated with residual lesion. Age (*P* = 0.16), menopause (*P* = 0.108), smoking (*P* = 0.409), diseases of the immune system (*P* = 0.998), parity (*P* = 0.349), gravidity (*P* = 0.805) and lesion level (*P* = 0.397) were not significant statistically associated with residual lesion (Table 2).

**Fig. 1:**
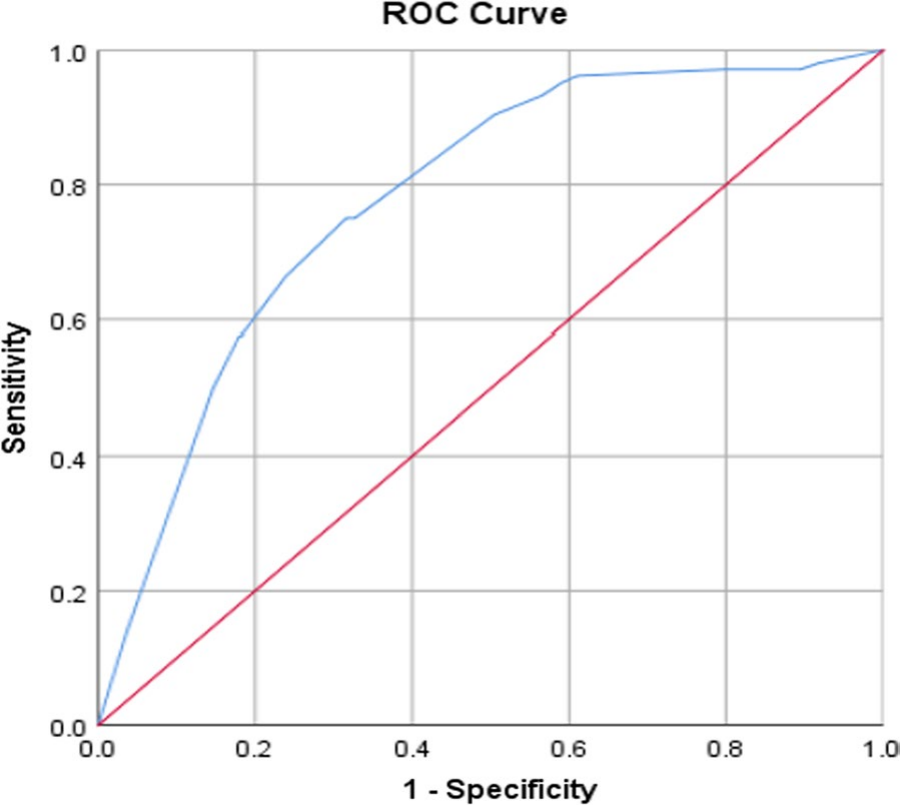
ROC curve for prediction of residual lesion in subsequent hysterectomy following CKC for HSIL. CKC: cold knife conization; HSIL: high-grade squamous intraepithelial lesion; ROC: receiver operator characteristic

Feasible variables that were found to be significantly associated with residual lesion on univariate analysis were entered into the logistic multiple factor regression equation. Logistic regression analysis revealed that positive margin (OR 4.015; 95% CI 2.526–6.381; *P* < 0.001), glandular involvement (OR 3.484; 95% CI 1.457–8.330; *P* = 0.005), HPV16/18 infection (OR 2.804; 95% CI 1.705–4.611; *P* < 0.001) and multiple HR-HPV infection (OR 1.813; 95% CI 1.130–2.909; *P* < 0.014) were independent risk factors for residual lesion (Table 3). The AUC calculated by logistic regression model wad 0.78, suggesting that its predictive value was good (Fig. 1).

**Table 3:** Multivariate analyses for prediction of residual lesion in subsequent hysterectomy specimen

Variables	B	SE	Wald	*P*-value	OR	95%CI
Margin positive	1.390	0.236	34.587	< 0.001*	4.015	2.526–6.381
Glandular involvement	1.248	0.445	7.877	0.005*	3.484	1.457–8.330
HPV-16/18 infection	1.031	0.254	16.516	< 0.001*	2.804	1.705–4.611
Multiple HR-HPV infection	0.595	0.241	6.091	0.014*	1.813	1.130–2.909

B, partial regression coefficient; SE, standard error; Wald, wald statistic; OR, odds ratio; CI, confidence interval

*Statistically significant

254 patients had positive resection margin, including 112 positive endocervical margin, 85 positive ectocervical margin and 57 positive combine margin. The rate of residual lesion in patients with positive margin was 27.6% (70/254), rate of residual lesion with positive endocervical margin was 31.3% (35/112), rate of residual lesion with positive ectocervical margin was 15.3% (13/85), rate of residual lesion with positive combine margin was 38.6% (22/57). There was significantly statistical difference in the residual lesions rate with positive endocervical margin, positive ectocervical margin and positive combine margin compared with negative margin (*P* < 0.05) (Table 4).

**Table 4:** Residual lesion rate in different site margin status after CKC

Parameter	Number of patienes		OR (95%CI)	*P-*value
	Total	Residual lesion		
Margin positive	254	70 (27.6%)	5.058 (3.244–7.885)	< 0.001*
Endocervical margin positive	112	35 (31.3%)	6.043 (3.556–10.268)	< 0.001*
Ectocervical margin positive	85	13 (15.3%)	2.4 (1.209–4.766)	0.01*
Combine margin positive	57	22 (38.6%)	8.356 (4.419–15.801)	< 0.001*
Margin negative	486	34 (7%)		

*CKC* cold knife conization, *OR* odds ratio, *CI* confidence interval

*Comparison with margin negative, statistically significant

In patients positive combined margin with glandular involvement positive and HPV-16/18 and multiple HR-HPV infection, the rate of residual lesion was 66.7% (6/9). In patients positive endocervical margin with glandular involvement positive and HPV-16/18 and multiple HR-HPV infection, the rate of residual lesion was 35.3% (6/17). In patients positive ectocervical margin with glandular involvement positive and HPV-16/18 and multiple HR-HPV infection, the rate of residual lesion was 23.1% (3/13) (Table 5).

**Table 5:** Rate of residual lesion with different risk factor combination

Condition	Probability of residual lesion
Positive combined margin and glandular involvement, and HPV16/18 and multiple HR-HPV infection	66.7% (6/9)
Positive endocervical margin and glandular involvement, and HPV16/18 and multiple HR-HPV infection	35.3% (6/17)
Positive ectocervical margin and glandular involvement, and HPV16/18 and multiple HR-HPV infection	23.1% (3/13)

*HR-HPV* High-risk human papillomavirus

## Discussion

Regarding the relevant guidelines on hysterectomy after CKC for HSIL, ⟪ Chinese Clinical Practice Guidelines for Gynecological Malignant Tumors (6th edition) ⟫ and ⟪2020 Chinese expert consensus for management of HSIL⟫ had made detailed and systematic explanations: repeated cervical conization with residual lesion in HSIL was difficult, HSIL combined with other gynecological diseases had surgical indications, patients with relapse and unable to perform conization after treatment, and patients with poor compliance after cervical conization, which could consider hysterectomy [6, 8]. This was the latest china guideline, to review the demographic and clinicopathological parameters of patients undergoing hysterectomy after CKC for HSIL in our hospital in recent 10 years, the following characteristics were found:the median age was 47 years, 34.3% were 50 years or above, and only 27.2% of whom were menopausal, indicating that elderly patients accounted for a small proportion in the population distribution. The proportion of the patients with education level below high school was as high as 78.5%, indicating that most of the patients receiving hysterectomy had low education level. There was also a slight difference in the administrative region, with 58% in rural areas. It can be preliminarily concluded that the decision of hysterectomy may be influenced by education level and economic status. Only 16.9% of patients needed surgical treatment for other gynecological diseases. In general, there were some differences between the characteristics of patients and latest guidelines. However, it was basically in line with 2006 edition of Chinese expert opinion. About the further management after conization, we strictly followed the Chinese expert consensus of the same period and informed the patients. we routinely signed the medical notification.

There was still a risk of residual and occult cervical cancer after conization for HSIL. In the study, we found that 14.1% patients after initial CKC had residual lesion on subsequent hysterectomy specimens. This finding was similar to the reported incidence of 5.4–60% in previous studies [3, 9–12]. In addition, the incidence of unrecognized invasive cervical cancer in this study (0.4%) was lower than the previously reported incidence (0.9–9.6%) [11–13], which may be related to the fact that patients with HSIL in our hospital were treated with CKC and the resection range was larger. As for the risk factors affecting residual lesion, previous literatures had shown that age, birth, menopausal status, lesion grade, margin status and lesion range were related to residual lesion after conization [13, 14]. In this study, we found that positive margin, positive glandular involvement, HPV16/18 and multiple HR-HPV infection were risk factors for residual lesion.

A large number of literatures had confirmed that positive margin was a predictor of residual lesion. If the cone margin was negative, the probability of residual lesion was about 2–24%, while the residual lesion rate with positive margin can be as high as 30–60% [15, 16]. In this study, the residual lesions rate was 27.6% in patients with positive margin and 7% with negative margin, indicating the residual lesion rate in patients with positive margin was more higher. Additionally, there were some differences in the residual rate of lesion with positive margin at different sites, and Chen found that patients with positive endocervical margin had a greater risk of residual lesion [17]. In this study, we found that the residual lesion rate of positive combine margin (38.6%) was higher than positive endocervical margin (31%) and ectocervical margin (15.3%), indicating that patients with positive combine margin need to pay more attention on residual lesion. All 3 patients with microinvasive carcinoma were positive combine margin. However, due to the limitation of the number of cases, the status of different margin was not grouped into the multi-factor regression model. Of course, positive margin did not mean treatment failure, and negative margin did not mean that follow-up review was not necessary. Simoes et al. [18] and Lubrano et al. [19] found that 60–80% of HSIL patients with positive margin had regression during follow-up. Chen found that the recurrence rate of patients with negative margin 24 months after surgery was 1.3%, the progress rate was 0.3% [17]. In this study, we found that the patients with negative margin still had a residual lesion rate of 7%. Therefore, long-term follow-up and management should still be adhered to after conization of HSIL.

Precancerous lesion of cervical cancer were mostly caused by persistent infection of HR-HPV. Single or multiple HR-HPV infection can increase the risk of cervical cancer, among which HPV16 and HPV18 were most closely related to cervical cancer. In this study, the infection rate of HR-HPV was as high as 93.2%, the infection rate of HPV16/18 was 55.1%, and the infection rate of multiple HR-HPV was 25.8%. It can be seen that HR-HPV infection plays an important role in the precancerous lesions of cervical cancer, and also reflects the importance of HPV screening. Other studies had shown that the sensitivity and specificity of HR-HPV in predicting CIN residual and recurrence were 91.0% and 83.8% respectively, which were independent risk factors for residual and recurrence of lesions [4, 12]. In this study, we found that HPV16/18 and multiple HR-HPV infection were risk factors for residual lesion after conization. Patients with HPV16/18 and multiple HR-HPV infection had 2.804 times and 1.813 times higher risk of residual lesion than those without HPV16/18 and multiple HR-HPV infection respectively. FAN found that the incidence of CIN III residue in HPV16 infection patients was significantly higher than that in other HR-HPV infected patients [20]. Wu et al. [21] found that the residual rate of multiple HR-HPV infection lesion was 43.1%, and multiple HR-HPV infection was a risk factor for residual lesion, which was consistent with the results of this study. Therefore, in the long-term follow-up and management of cervical lesions after treatment, HR-HPV testing was an important follow-up means.

There were many controversies about the correlation between glandular involvement and residual lesion. Jeong-yeol Park found that among patients after CIN III conization, glandular involvement was not a risk factor for residual lesions [11]. Chien-Hsing Lu found positive glandular involvement was a risk factor for residual lesions in the univariate analysis of CIN III, while it was not a risk factor in the multivariate analysis [22]. In this study, we found that the rate of residual lesion was significantly higher in patients with positive glandular involvement than in those without glandular involvement, and that positive glandular involvement was an independent risk factor for residual lesion. This may be due to the higher degree of cell proliferation and invasion ability of patients with CIN involving glands, and the abnormal cells were mostly hidden in the cervical duct gland covered by normal epithelium, resulting in residual recurrence of lesions or progression to higher-grade lesions, or even invasive cancer.

According to the multiple analyses of risk factors for residual lesion, we found the rate of residual lesion was 66.7% in which patients positive combined margin with glandular involvement positive and HPV-16/18 and multiple HR-HPV infection. However, neither risk factors nor suspected residual lesions were indications for hysterectomy. For patients with unclear indications of hysterectomy, long-term standardized follow-up management should be recommended. Repeated cervical conization was preferred if there was evidence of residual HSIL lesions during follow-up [6, 8]. At the same time, we will also take into consideration the actual situation of patients, such as patients' fear emotion, poor economic conditions, and poor follow-up compliance.

This study had several limitations. First, this was a single-center retrospective study, and the reliability of the reported data was reduced. Second, the small sample size makes it impossible to accurately evaluate the correlation between the status of different margin and residual lesions. Third, HPV testing is only qualitative detection, not the detection of the amount of virus vector, the correlation between HPV infection and residual lesions was less convincing.

In conclusion, positive margin, positive glandular involvement, HPV16/18 and multiple HR-HPV infection were high risk factors of residual lesions for HSIL after CKC. Appropriate application of these high-risk factors, comprehensive development of standardized based on the individual treatment plan, in order to achieve accurate treatment.

## Data Availability

The datasets generated and analysed during the current study are not publicly available due avoid unreasonable use by third parties or organizations, but are available from the corresponding author on reasonable request.

## Abbreviations

CKC: Cold knife conization
HSIL: High-grade squamous intraepithelial lesion
LEEP: Loopelectrical excision procedure
CAP: American Society for Pathology
ASCCP: American Society for Colposcopy and Cervical Pathology
CIN: Cervical intraepithelial neoplasia
HR-HPV: High-risk human papillomavirus
TCT: Thin-cytologic test
ROC: Receiver operator characteristic
AUC: Area under receiver operator characteristic curve
